# *Sept8/SEPTIN8* involvement in cellular structure and kidney damage is identified by genetic mapping and a novel human tubule hypoxic model

**DOI:** 10.1038/s41598-021-81550-8

**Published:** 2021-01-22

**Authors:** Gregory R. Keele, Jeremy W. Prokop, Hong He, Katie Holl, John Littrell, Aaron W. Deal, Yunjung Kim, Patrick B. Kyle, Esinam Attipoe, Ashley C. Johnson, Katie L. Uhl, Olivia L. Sirpilla, Seyedehameneh Jahanbakhsh, Melanie Robinson, Shawn Levy, William Valdar, Michael R. Garrett, Leah C. Solberg Woods

**Affiliations:** 1 The Jackson Laboratory, Bar Harbor, ME USA; 2 HudsonAlpha Institute, Huntsville, AL USA; 3 Department of Pediatrics and Human Development, Department of Pharmacology, Michigan State University, Grand Rapids, MI USA; 4 Departments of Pediatrics and Physiology, Medical College of Wisconsin, Milwaukee, WI USA; 5 Department of Internal Medicine, Wake Forest School of Medicine, Winston-Salem, NC USA; 6 Department of Genetics, University of North Carolina at Chapel Hill, Chapel Hill, NC USA; 7 Lineberger Comprehensive Cancer Center, University of North Carolina at Chapel Hill, Chapel Hill, NC USA; 8 Department of Pharmacology, Medicine (Nephrology), Pediatrics (Genetics), University of Mississippi Medical Center, Jackson, MS USA; 9 Department of Pathology, University of Mississippi Medical Center, Jackson, MS USA

**Keywords:** Genetics, Systems biology

## Abstract

Chronic kidney disease (CKD), which can ultimately progress to kidney failure, is influenced by genetics and the environment. Genes identified in human genome wide association studies (GWAS) explain only a small proportion of the heritable variation and lack functional validation, indicating the need for additional model systems. Outbred heterogeneous stock (HS) rats have been used for genetic fine-mapping of complex traits, but have not previously been used for CKD traits. We performed GWAS for urinary protein excretion (UPE) and CKD related serum biochemistries in 245 male HS rats. Quantitative trait loci (QTL) were identified using a linear mixed effect model that tested for association with imputed genotypes. Candidate genes were identified using bioinformatics tools and targeted RNAseq followed by testing in a novel in vitro model of human tubule, hypoxia-induced damage. We identified two QTL for UPE and five for serum biochemistries. Protein modeling identified a missense variant within *Septin 8* (*Sept8)* as a candidate for UPE. *Sept8/SEPTIN8* expression increased in HS rats with elevated UPE and tubulointerstitial injury and in the in vitro hypoxia model. SEPTIN8 is detected within proximal tubule cells in human kidney samples and localizes with acetyl-alpha tubulin in the culture system. After hypoxia, SEPTIN8 staining becomes diffuse and appears to relocalize with actin. These data suggest a role of SEPTIN8 in cellular organization and structure in response to environmental stress. This study demonstrates that integration of a rat genetic model with an environmentally induced tubule damage system identifies *Sept8/SEPTIN8* and informs novel aspects of the complex gene by environmental interactions contributing to CKD risk.

## Introduction

The onset and progression of chronic kidney disease (CKD) is often related to pre-existing hypertension and diabetes, and those with CKD are at a significant risk to develop other cardiovascular diseases^1,2^. The vast majority of CKD is complex with a significant contribution from genetics; however, environmental influences also play an essential role in adult CKD^3^. To date, human genome wide association studies (GWAS) have identified hundreds of single nucleotide polymorphisms (SNPs) associated with CKD in the context of hypertension, diabetes, and obesity, but in total the effect of identified genetic variation still explains only a small proportion of the heritable variance, and many of these associations lack functional validation^4^.

The genetic dissection and validation of these associations has been challenging in humans, requiring additional model systems to provide insight into gene function. In particular, inbred rat models have been critical in determining CKD genetic mechanisms, serving as the first genetic model of epistasis and establishing independence of renal damage from hypertension^5,6^. Multiple genes have overlapping variant associations with kidney damage in both rat and human including *SHROOM3*, *RAB38*, *SH2B3*, and *PLEKHA7* and *ARHGEF11*^7–12^. These studies show a synergistic nature of both glomerular and tubule genetics in proteinuria and CKD. The initial glomerular alterations that lead to proteinuria alter the tubule component through factors such as hypoxia which then advance into fibrosis and CKD^13,14^.

Here, we have used an alternative to traditional rodent genetic studies by employing the heterogeneous stock (HS) rat model to identify genetic factors involved in kidney disease. A strength of the HS model is that it allows for genetic fine-mapping to only a few megabases, reducing the number of candidate genes and simplifying gene identification^15^. HS rats were created by interbreeding eight inbred founder strains and maintaining the colony to minimize inbreeding^16^. We have previously used HS rats to fine-map genetic loci and identify underlying causal genes for diabetes^17–19^ and adiposity^20,21^, and have demonstrated phenotypic variation for multiple kidney traits^22^. In the current study, we identified genomic loci for urinary protein excretion (UPE) and intermediary serum biochemistries associated with CKD, leading to *Septin 8 (Sept8)* as a candidate gene linked with renal injury. We demonstrate that increased levels of *Sept8* are associated with renal damage in the rat model and use a unique in vitro renal tubule hypoxia model to demonstrate a potential role of *SEPTIN8* in cellular structure and organization in response to hypoxia induced stress.

## Methods

### Animals

Heterogeneous stock colony: The NMcwi:HS colony, hereafter referred to as HS, was initiated by the NIH in 1984 using the following eight inbred founder strains: ACI/N, BN/SsN, BUF/N, F344/N, M520/N, MR/N, WKY/N and WN/N^16^. Rats used for the current study were obtained from a colony maintained at the Medical College of Wisconsin (MCW) after approximately 70 generations of breeding. Rats were housed two per cage in micro-isolation cages in a conventional facility using autoclaved bedding (sani-chips from PJ Murphy). Animals used for this study were caged under controlled temperature, humidity, and 12-h light/12-h dark conditions. They had ad libitum access to autoclaved Teklad 5010 diet (Harlan Laboratories) and were provided reverse osmosis water chlorinated to 2–3 ppm. We tested 425 HS male rats in the following protocol, 245 of which were genotyped for GWAS. This group of animals is from a subset of HS rats previously studied to map loci associated with body weight and obesity related traits^21^.

### Phenotyping protocol

At 24 weeks of age, animals were placed into metabolic cages for 24 h with free access to water, and UPE was determined as previously described^23^. After removal from the metabolic cage, animals were euthanized by decapitation and fasting serum and multiple tissues were collected. Serum clinical chemistries, including measures of renal function (creatinine and blood urine nitrogen), lipid metabolism (total cholesterol, HDL, LDL, and triglycerides), and electrolytes (sodium, potassium, calcium, and chloride) were performed using an automated chemistry analyzer as done previously^24^. All protocols were approved by the IACUC committee at MCW and all methods were performed in accordance with relevant guidelines and regulations. The studies were carried out in compliance with the ARRIVE guidelines (https://arriveguidelines.org/). Phenotyping data have been deposited in RGD (www.rgd.mcw.edu).

### Histology analysis of HS rat kidneys

Kidneys were fixed in 10% buffered formalin, embedded in paraffin, cut into 4-μm sections and stained with Periodic acid Schiff (PAS) and/or Masson’s trichrome on selected HS animals striated by very low (n = 10), low (n = 10), moderate (n = 10), and high UPE (n = 8). For glomeruli, morphometric analysis [diameter (um) and area (um2)] was performed on 20 randomly selected images (PAS at 40X) per section. Tubulointerstitial injury was determined by evaluation of slides stained with Masson’s Trichrome to quantify the percent fibrosis (blue staining) compared to background in 20 randomly selected images from renal cortex as previously done^25^. Tubulointerstitial injury was evaluated separately on a semi-quantitative scale from 0 (normal) to 4 (severe) using a minimum of 20 randomly selected images (Masson’s Trichrome at 20 X) as follows: grade 0, no changes; grade 1, mild tubule atrophy/fibrosis involving less than 25%; grade 2, lesions affecting 25–50%; grade 3, lesions affecting 50–75%; and grade 4, lesions affecting > 75%^25^. Morphometric analysis was also used to measure vessel wall thickening (PAS at 40X). Vessel wall thickening (vessel media, um2) was calculated by measuring the outer circumference of the vessel minus the inner circumference of the lumen (20 random images at 40X per HS rat). All measurements were done blinded to sample groups. Images were captured using SeBaP4-PH1 Brightfield/Phase contrast microscope (Laxco, Mill Creek, WA) and analyzed using Nikon Elements image analysis software.

### Genotyping

HS rat DNA was extracted from tail tissue using either the Qiagen DNeasy kit (Valencia, CA) or a phenol–chloroform extraction. 245 HS rats were genotyped using the Affymetrix GeneChip Targeted Genotyping technology on a custom 10 K SNP array panel as previously described^26^, with marker locations based on rat genome assembly 6.0. Samples were genotyped by HudsonAlpha Institute (http://hudsonalpha.org). From the 10,846 SNPs on the array, 8,218 were informative and produced reliable genotypes in the HS rats. From these final informative markers, the average SNP spacing was 284 Kb, with an average heterozygosity of 25.68%.

### Targeted RNAseq

Expression analysis was performed on select candidate genes residing within identified loci using a next generation sequencing approach as previously described^27^. RNA was isolated from kidney collected from most of the HS rats that had both phenotype and genotype information (n = 240) using an automated KingFisher Flex nucleic acid system along with KingFisher Pure RNA Kit. RNA was evaluated for quantity (Nanodrop One and Qubit Fluorimeter) and quality using Qiagen QIAxcel advanced system. The Illumina DesignStudio application (http://designstudio.illumina.com/) was utilized to design custom amplicons across exon–intron boundaries of target genes (n = 36 gene with 1–2 probes per gene). The gene target/probes that were designed/used are listed in Supplemental Table S1. Based on the DesignStudio output, the TruSeq Targeted RNA Custom Panel Kit was ordered and subsequently utilized to prepare libraries for collected RNA samples. The Illumina MiSeq platform allows for analysis of pooled libraries (e.g., n = 96–384 RNA samples) to be processed at a single time as individual samples will have a unique “barcode.” Libraries were sequenced on Illumina MiSeq using MiSeq Reagent Kit v2 (150 cycle). Sequencing reads were de-multiplexed and aligned to rn6 genome assembly using RNA Amplicon Application (along with custom panel manifest) available on Illumina BaseSpace Computing Platform (http://basespace.illumina.com/). Aligned reads for each gene were normalized to count per million for downstream analysis.

### Statistical analysis

#### Heritability estimation for kidney and biochemistry traits

Prior to analysis, all phenotypes were normalized using a rank-based inverse normal transformation, and additionally scaled to have mean 0 and standard deviation 1. Narrow-sense heritability was estimated for each transformed phenotype using a Bayesian linear mixed model (LMM) using INLA^28,29^ as in^21^. Briefly, the LMM included a random “polygenic” effect, representing the effect of overall relatedness (calculated using^30^). Heritability, ${h}^{2}$, was defined as the proportion of variance attributed to polygenic effects vs residual noise,

1 $$h^{2} = \frac{{\tau_{\text{poly}}^{2} }}{{\tau_{\text{poly}}^{2} + \sigma ^{2} }},$$

where $\tau_{\text{poly}}^{2}$ is polygenic effect variance and $\sigma^{2}$ is the residual noise variance. The inverses of the variance components, $\tau_{\text{poly}}^{ - 2}$ and $\sigma^{ - 2} ,$ were given Gamma (1,1) priors to ensure that the prior on $h^{2}$ was uniform between 0 and 1. For all other settings, the defaults of INLA were used.

#### Genome-wide association

As previously described, quantitative trait loci (QTL) were identified by genome-wide association of imputed allele dosages of genotyped SNPs^21^. A hidden Markov model^31^ was used to infer each HS rat’s haplotype mosaic and thereby obtain robust estimates of each SNP’s genotype. Association tests were then performed, SNP-by-SNP, on each trait using a likelihood ratio test from the LMM described earlier but with an added SNP effect term in the alternative model. Tests of the SNP effect yielded *p* values that are reported on the negative log to the base 10 scale, or “logP”. Genome-wide significance thresholds for logP scores were estimated using 1000 parametric bootstrap samples from the fitted null model^17,32^. Linkage disequilibrium (LD) intervals for the detected QTL were defined by the window of neighboring markers that met a set level of LD, measured with the squared correlation coefficient r^2^; we used r^2^ ≥ 0.5.

#### Founder haplotype effect estimation at detected QTL

To characterize each QTL signal, we used the Diploffect model^33^ [https://github.com/gkeele/Diploffect.INLA], which estimates the relative contributions of alternate founder haplotypes. Diploeffect is a Bayesian hierarchical approach designed to work with probabilistically inferred haplotype descent, providing shrinkage that mitigates instability from low haplotype frequencies. In addition to the population structure effect in Eq. (1), it models two genetic components at the QTL: additive (haplotype) effects, ie the effect of each dose of haplotype (eg, WKY); and dominance deviations from the additive model for specific heterozygous combinations of haplotype, (eg, WKY-ACI). Dominance deviations are typically less informed, representing potentially 28 heterozygous states, but their inclusion stabilizes additive effect estimation. Both have their own variance parameters, ${\tau}_{\text{add}}^{2}$ and ${\tau}_{\text{dom}}^{2}$, with QTL effect size recorded as the intraclass correlation coefficient

2 $${h}_{\text{QTL}}^{2}= \frac{{\tau}_{\text{QTL}}^{2}}{{\tau}_{\text{QTL}}^{2}+{\tau}_{\text{poly}}^{2}+ \sigma^{2}}$$

where ${\tau}_{\text{QTL}}^{2}= {\tau}_{\text{add}}^{2}+ {\tau}_{\text{dom}}^{2}$. The model was fitted using 200 importance samples from INLA^28^, with the same phenotype transformations and variance component priors as used with heritability estimation, described above.

### Bioinformatic analysis and protein modeling

HS founder sequence (www.rgd.mcw.edu; genome build Rn6) was used to identify highly conserved, non-synonymous coding variants within each QTL that were predicted to be damaging by Polyphen (http://genetics.bwh.harvard.edu/pph/) and/or SIFT (https://sift.bii.a-star.edu.sg/), focusing on variants specific to founder strains that showed non-zero haplotype effects at the locus. High probability variants were confirmed using Sanger sequencing and then analyzed in the Sequence-to-Structure-to-Function analysis as previously described^34^. Briefly, proteins were assessed with codon selection analysis of multiple species open reading frames, inspected for linear motif impact near variants of interest, and modeled with I-TASSER^35^ and YASARA^36^. Models were then assessed for likely impact on protein folding and/or function based on model confidence, phylogenetic sequence alignment, conservation, and whether or not the variant altered structural packing, molecular dynamic simulations, binding partners, linear motifs or post-translational modifications.

### Mediation analysis

Expression levels of local genes were evaluated as potential mediators of the two UPE QTL through mediation analysis^37^, previously employed in HS rats^21^. Genes were defined as local if they were located within the QTL LD interval. Briefly, to declare mediation, we examined the relationships underlying detected QTL based on four sequential criteria. (1) A QTL was detected: this established a causal relationship between genetic variation and UPE that may involve gene expression intermediates. (2) The potential mediator possessed a cis-eQTL at the UPE QTL: this established that the eQTL co-localizes with the QTL. To avoid false signals, mediators were required to have greater than 25% non-zero expression across all samples and the false discovery rate (FDR) of mediator eQTL *p* values was controlled (Benjamini-Hocheberg) at 0.1; genome-wide multiple testing correction was unnecessary because testing was constrained to the QTL for local genes. (3) After conditioning on the candidate mediator, the significance of the QTL was greatly reduced, consistent with full mediation. Local genes with cis-eQTL were evaluated as potential full mediators of the QTL by fitting the QTL regression models (alternative and null) with the gene expression mediator included as a covariate. If the resulting *p* value was greater than 0.05 then full mediation was considered plausible. (4) Evidence supporting partial mediation was observed, in which the mediator provides additional information beyond the QTL. This criterion was formally tested for the expression of local genes with cis-eQTL by comparing the QTL model with mediator included as a covariate to the QTL model without the mediator. Similar to step 2, significant partial mediators were called based on having FDR q-values ≤ 0.1. Genes that satisfied step 2 and steps 3 and/or 4 would be declared candidate mediators of the QTL.

### RPTEC-TERT1 hypoxia model

Renal proximal tubule epithelial cells (RPTEC) immortalized with TERT1 (ATCC, CRL-4031) were grown to confluency in 6-well plates using DMEM:F12 supplemented with RPTEC growth kit (ATCC) at 5% CO_2_. Once cells reached confluency, they were placed at 75 RPM using a MaxQ CO_2_ plus shaker for 1 day followed by elevation to 150 RPM for 4 weeks. Although we used cells cultured under this shaking condition for 4 weeks for the current study, a time course of morphological changes suggests that approximately 2 weeks of shaking yields the same level of cellular change. We have also determined that the cells can be maintained under shaking for up to 6 months or longer. Control cells were treated the same as shaking cells except they were held static in the same incubator. Media was changed every other day on all cultures. Hypoxia was induced on cultures (shaking and static) within a PHCbi incubator to 1% O_2_ using nitrogen replacement for 48 or 96 h, with a media change at 48 h in the 96 h experiment. Morphometric analysis was conducted using 40 × light images generated on the Evos and processed through MorphoLibJ^38^ in ImageJ using morphological segmentation set to border image with a watershed segmentation tolerance of 17. We demonstrate that shaking, in combination with hypoxia, leads to a cellular damage state in vitro.

### RPTEC-TERT1 RNAseq

RNA was extracted using RNeasy plus (Qiagen) followed by quantification using Qubit RNA High Sensitivity assay and quality assessed using a Fragment Analyzer RNA Kit. Four groups were studied: 20% O_2_ static (n = 3), 20% O_2_ shaking (n = 2), 1% O_2_ static (n = 2), and 1% O_2_ shaking (n = 2). RNA was sent to Novogene for library prep and sequencing. In short, PolyA capture RNA libraries were generated and sequenced for paired end 150 cycle reads. FASTQ files were then assessed with a quasi-aligner onto the human Ensembl 96 all transcript release using Salmon^39^. Log2 fold change (FC) was calculated for either 20% shaker vs static or 20% shaker vs 1% shaker and a t-test calculated for each comparison, focusing on unique transcripts instead of genes. Cutoff levels for differentially expressed genes in each group for fold change was > 2 or < -2 and a *p* value < 0.05. Gene, pathway, and protein–protein interaction enrichment was performed using STRING^40^. Data files are submitted to NCBI as BioProject PRJNA604721 for BioSample SAMN13979617 with reads available in the SRA (SRR11014348, SRR11014347, SRR11014346, SRR11014345, SRR11014344, SRR11014343, SRR11014342, SRR11014341, SRR11014340).

### RPTEC-TERT1 immunofluorescence

Cells were prepared for Immunofluorescence using the Image-iT kit (ThermoFisher). Primary antibodies were incubated overnight in 3% BSA at 1:200. These included ZO-1 (ThermoFisher, #33-9100), Acetyl-alpha Tubulin (ThermoFisher, #32-2700), and SEPTIN8 (ThermoFisher, # PA531356). Secondary antibodies (1:1000) against mouse (ThermoFisher, #A32723) or rabbit (ThermoFisher, #A32740) and actin stain (Alexa Fluor 647 Phalloidin) were incubated in 3% BSA for 1 h. Following PBS wash of cells, the nuclei were stained with Nucblue fixed cell stain (ThermoFisher, # R37606). Images of cells were generated on an EVOS FLoid for fluorescence and EVOS XL for light images.

### Immunohistochemistry of human kidney biopsies

To determine if SEPTIN8 is expressed in human kidney, we assessed its levels in human kidney biopsies with varying levels of damage. Human kidney biopsies were collected with approval by the Institutional Review Board (DHHS FWA #00003630, IRB 2 #00005033) at the University of Mississippi Medical Center (Protocol 2010-0297). All methods were carried out in accordance with relevant guidelines and regulations. A waiver of consent was granted as minimal risk and only de-identified samples were collected per IRB expedited review and category exemption 5. Immunohistochemistry was performed as previously done^41^ using unstained sections and primary antibodies directed at SEPTIN8 (ThermoFisher, # PA531356) and detected by DAB (Ultravision LPValue Detection System, Thermo Scientific). Slides were counterstained with Methyl green. Images were captured using SeBaP4-PH1 Brightfield/Phase contrast microscope (Laxco, Mill Creek, WA). SEPTIN8 staining was evaluated in human kidney biopsy material from 3 patients with various etiologies, as evaluated by a renal pathologist. These included control biopsy 22 (few small loci of inflammation, otherwise unremarkable pathology), collected after nephrectomy subsequent to renal pelvic carcinoma; biopsy12 (hematuria, slight interstitial fibrosis, tubular necrosis, and non-nephrotic range proteinuria); and biopsy 4 (diabetic mild glomerulosclerosis, moderate diffuse interstitial fibrosis, with tubular atrophy and tubular necrosis). All samples were run at the same time and were incubated with DAB for the same amount of time.

## Results

### HS urinary protein excretion (UPE) genetic mapping

**Figure 1:**
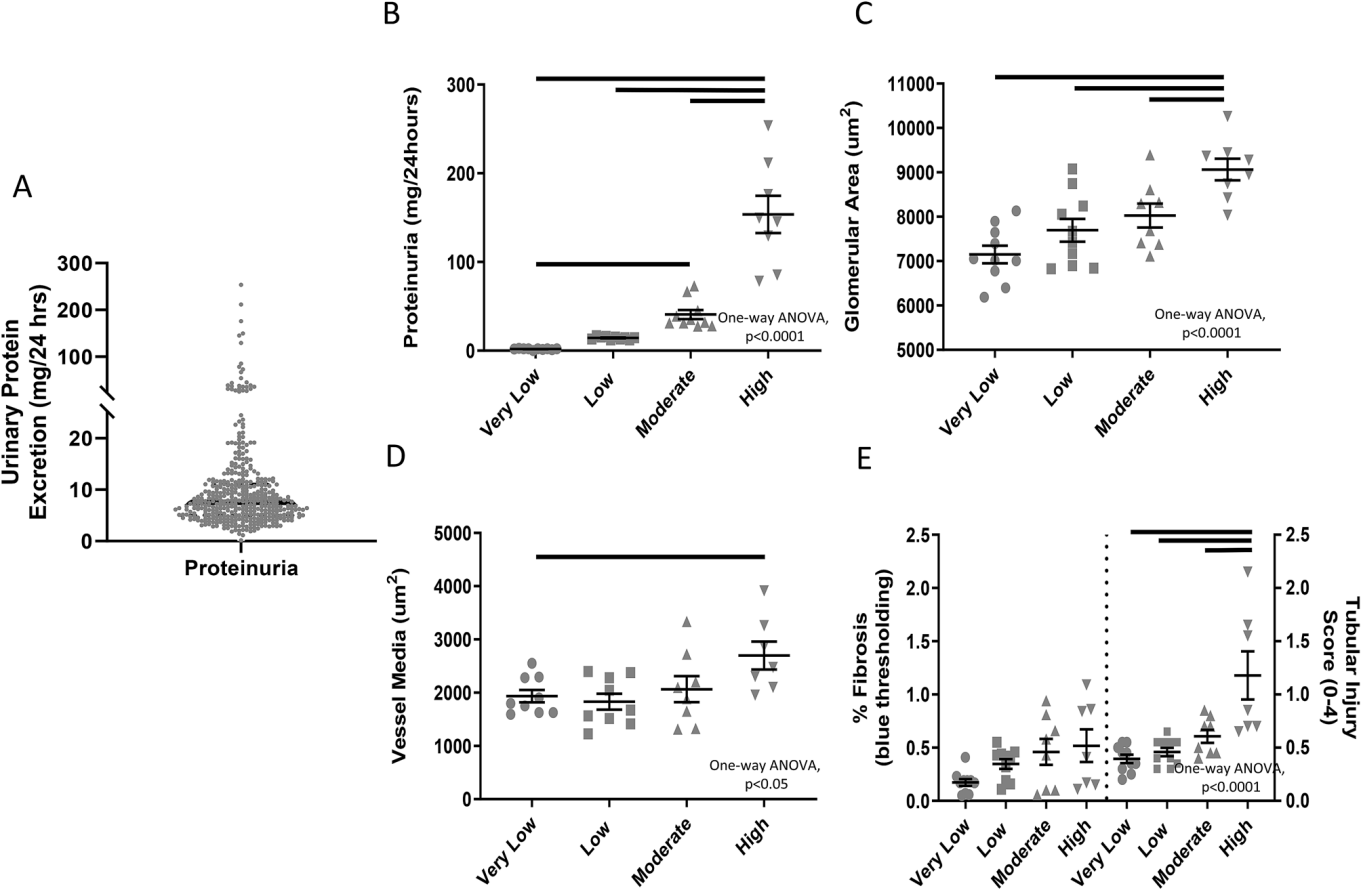
Distribution of urinary protein excretion (UPE) and the relationship between UPE and histological analysis of the kidney in HS rats. (**A**) 24-week old HS rats (n = 425) exhibit strong variation in UPE (mg/24 h). The thick black horizontal line indicates the median with the dashed lines denoting 25% and 75% quartiles, respectively. (**B**) UPE is shown for a sub-set of rats selected for histological analysis from one of four groups: very low, low, moderate, and high UPE. Histological analysis demonstrates that there is a statistically significant increase in (**C**) glomerular area and (**D**) vessel hypertrophy in rats with high proteinuria. (**E**) Although HS rats exhibit relatively limited fibrosis, those with high proteinuria exhibit significantly higher scores of tubular injury. Solid horizontal bars represent statistically significant differences based on a one-way ANOVA (*p* < 0.0001), the black lines above the bar graph denote *p* < 0.05 between each group.

HS rats exhibited large variation in UPE levels with a mean of 11.8 mg/day and a range of 0.166 to 660.9 mg/day by 24 weeks of age (Fig. 1A). Consistent with previous findings, 16 out of 245 (6.5%) of the HS rats exhibited UPE greater than 20 mg/day and are considered to have pathological levels typically noted as proteinuria^22^. Four rats (1.6%) had a single kidney, two of which had normal UPE and two with proteinuria. Select animals from the population striated by very low, low, moderate, or high UPE demonstrated a significant increase in glomerular diameter and vessel hypertrophy with increasing levels of proteinuria (one-way ANOVA, *p* < 0.0001) (Fig. 1B–D). Animals with high proteinuria (153 ± 21.1 mg/24 h) demonstrated a 26% increase in glomerular size compared to the very low proteinuria group (1.7 ± 0.2 mg/24 h) (Fig. 1C, Figure S1). We also found a significant correlation (r = 0.61, *p* < 0.0001) between UPE and glomerular area. Similar changes were seen in vessel thickening (Fig. 1D). Although there were relatively low levels of fibrosis (measured by Masson’s Trichrome staining), semi-quantitative analysis of tubular and interstitial injury was significantly increased with increasing levels of UPE (one-way ANOVA, *p* < 0.0001; Fig. 1E, Figure S1). UPE was strongly influenced by genetics with heritability of 0.45 (0.25–0.55; Table 1).

**Table 1:** Narrow sense heritability for urinary protein and serum biochemistries.

Trait	Heritability: posterior mode (95% highest posterior density interval)
Urinary protein	0.45 (0.25–0.55)
Albumin	0.58 (0.33–0.75)
Alk phosphatase	0.60 (0.35–0.76)
ALT	0.59 (0.32–0.77)
AST	0.30 (0.16–0.53)
BUN	0.39 (0.20–0.63)
Calcium	0.39 (0.19–0.63)
Chloride	0.46 (0.24–0.68)
HDL cholesterol	0.83 (0.64–0.90)
LDL cholesterol	0.84 (0.69–0.91)
Total cholesterol	0.82 (0.67–0.89)
CO_2_	0.44 (0.23–0.67)
Creatinine	0.66 (0.38–0.82)
Glucose	0.63 (0.41–0.79)
Phosphorus	0.52 (0.28–0.71)
Potassium	0.36 (0.18–0.59)
Sodium	0.33 (0.16–0.57)
Bilirubin	0.35 (0.17–0.61)
Protein	0.69 (0.44–0.82)
Triglycerides	0.44 (0.22–0.67)

**Figure 2:**
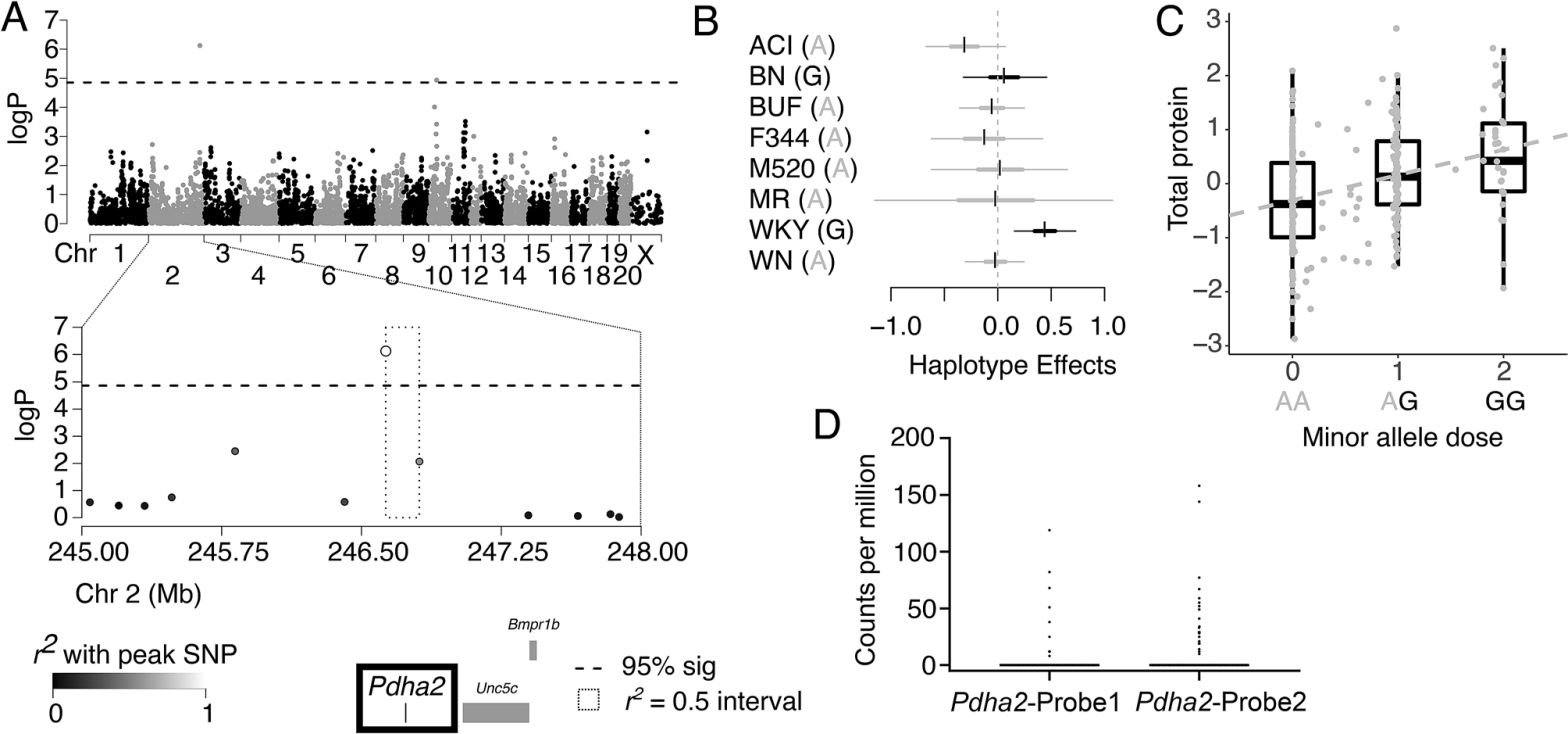
Genome-wide scan and founder effects at the chromosome 2 QTL for UPE. (**A**) Genome scan for UPE shows significant loci on chromosomes 2 and 10. The x-axis is the position on the chromosome, and the y-axis is the − log_10_P level of association. Genome-wide significance thresholds were calculated using parametric bootstraps from the null model. Linkage disequilibrium support interval (highlighted as a dashed line box) of the chromosome 2 locus is only 0.18 Mb and contains a single gene, *Pdha2* (bold). (**B**) Additive haplotype effects were estimated using the Diploffect model, taking into account uncertainty in haplotype state. Single-nucleotide polymorphism (SNP) allele information is overlaid on the haplotype effects, with minor allele colored black and major colored gray. The WKY haplotype significantly increases urinary protein at this locus. (**C**) Effect of genotype at the peak marker on urinary protein reveals that the G allele is associated with increased urinary protein. Boxplots represent data categorized by most likely genotype. (**D**) Targeted RNAseq data demonstrating very limited expression of *Pdha2* in the kidney using two separate probes. These data, together with a lack of potentially damaging variants in this gene and lack of support from mediation analysis, suggest that *Pdha2* is not the causal gene at this locus.

**Figure 3:**
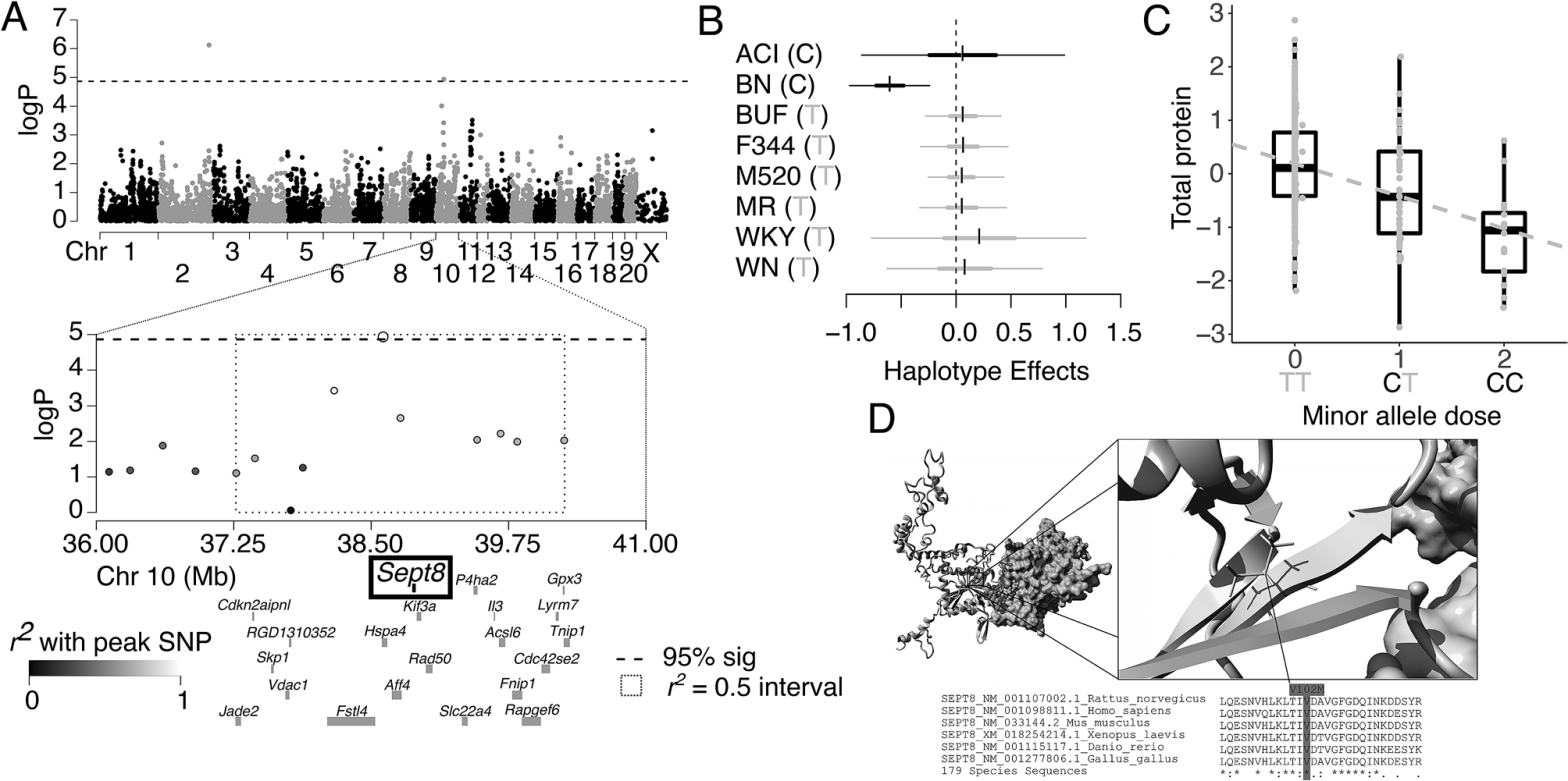
Genome-wide scan and founder effects at the chromosome 10 QTL for UPE followed by protein modeling of SEPTIN8. (**A**) Genome scan for urinary protein shows significant loci on chromosomes 2 and 10. The x-axis is the position on the chromosome, and the y-axis is the − log_10_P level of association. Genome-wide significance thresholds were calculated using parametric bootstraps from the null model. Linkage disequilibrium support interval (highlighted as a dashed line box) for the chromosome 10 locus is 2.99 Mb. Annotation of a sub-set of the 50 genes in this region are shown below the plot. (**B**) Additive haplotype effects were estimated using the Diploffect model, which takes into account uncertainty in haplotype state. Single-nucleotide polymorphism (SNP) allele information is overlaid on the haplotype effects, with minor allele in black and major allele in gray. The BN haplotype significantly decreases urinary protein at this locus, with high uncertainty from the ACI haplotype. Both BN and ACI share the C allele at this locus. (**C**) Effect of genotype at the peak marker on urinary protein showing that the C allele decreases urinary protein. (**D**) Protein modeling for SEPTIN8. Variant V102M of SEPTIN8 falls within a hydrophobic core near the SEPTIN8 dimer contacts. A zoomed in view is shown to the right. The bottom panel shows sequence alignments of amino acids in multiple species. SEPTIN8 amino acid 102 is 100% conserved as a V throughout 179 species.

Two significant QTL were identified for UPE (mg/day): chromosome 2 (246.63–246.81 Mb, − logP = 6.1) and chromosome 10 (37.27–40.26 Mb, − logP = 4.9, Figs. 2A, 3A) with effect sizes of 20% and 19%, respectively. The chromosome 2 locus contained only a single gene, *Pdha2.* The G allele (driven mainly by the WKY haplotype) was associated with an increase in UPE at this locus (Fig. 2B,C). Targeted RNAseq demonstrated that *Pdha2* expression was essentially undetectable in total kidney from most samples (Fig. 2D), consistent with a known testis-specific expression pattern (based on several expression databases). Although the limited expression in kidney did appear to map as a cis-eQTL, *Pdha2* expression levels did not correlate with UPE levels and statistical mediation analysis (see methods) did not support *Pdha2* expression as a mediator of the UPE QTL. In addition, the HS founders did not possess any sequence variants within *Pdha2*. Expression levels were determined for several other genes just outside the UPE QTL (*Bmpr1b, Pdlim5, Stpg2* and *Unc5c*) and none map cis-eQTL or correlate with UPE. Furthermore, none of these genes possessed non-synonymous or loss of function variants. This evidence suggests that a variant within the chromosome 2 locus may be regulating expression of a gene outside of this region.

The chromosome 10 locus contained 50 genes. The C allele, possessed by ACI and BN, was associated with decreased UPE (Fig. 3B,C). This effect was driven mainly by the BN haplotype, with few rats possessing the ACI haplotype, reflected in the wide estimate intervals (Fig. 3B). We measured expression levels of 31 of the 50 genes in the region (Supplemental Table S1), with 24 showing high levels of expression in the kidney. Three of the genes had strong cis-eQTL detected in the chromosome 10 locus: *Cdk13, Kif3a* and *P4ha2*. Mediation analysis, however, did not support a role of these genes’ expression in regulating UPE at the QTL. Seven genes in this region harbored missense variants in the founder strains that carry the predisposing allele (ACI and/or BN), with two of these variants (*Sept8* and *Rapgef6*) having a potential functional role. *Rapgef6* had a non-synonymous variant (D1123E) in the BN strain that fell on a site with some selective pressure, although without complete evolutionary conservation, thus unlikely to impact protein function. Within *Sept8*, the ACI founder harbored an A at position 38,891,224 bp whereas all other strains harbor a G, resulting in the amino acid substitution V102M. The variant fell on a highly conserved site with altered function predicted by PolyPhen2 and SIFT. Based on protein modeling, this variant fell within a hydrophobic core near the SEPTIN8 dimer contacts and is 100% conserved as a V throughout 179 species (Fig. 3D). The protein modeling and QTL analysis both support that genetic variation in *Sept8*, specifically the V102M mutation, was associated with altered renal injury. Specifically, the C allele of the detected QTL (from BN and ACI), protected against high UPE.

### Development of a human tubule hypoxia damage model

**Figure 4:**
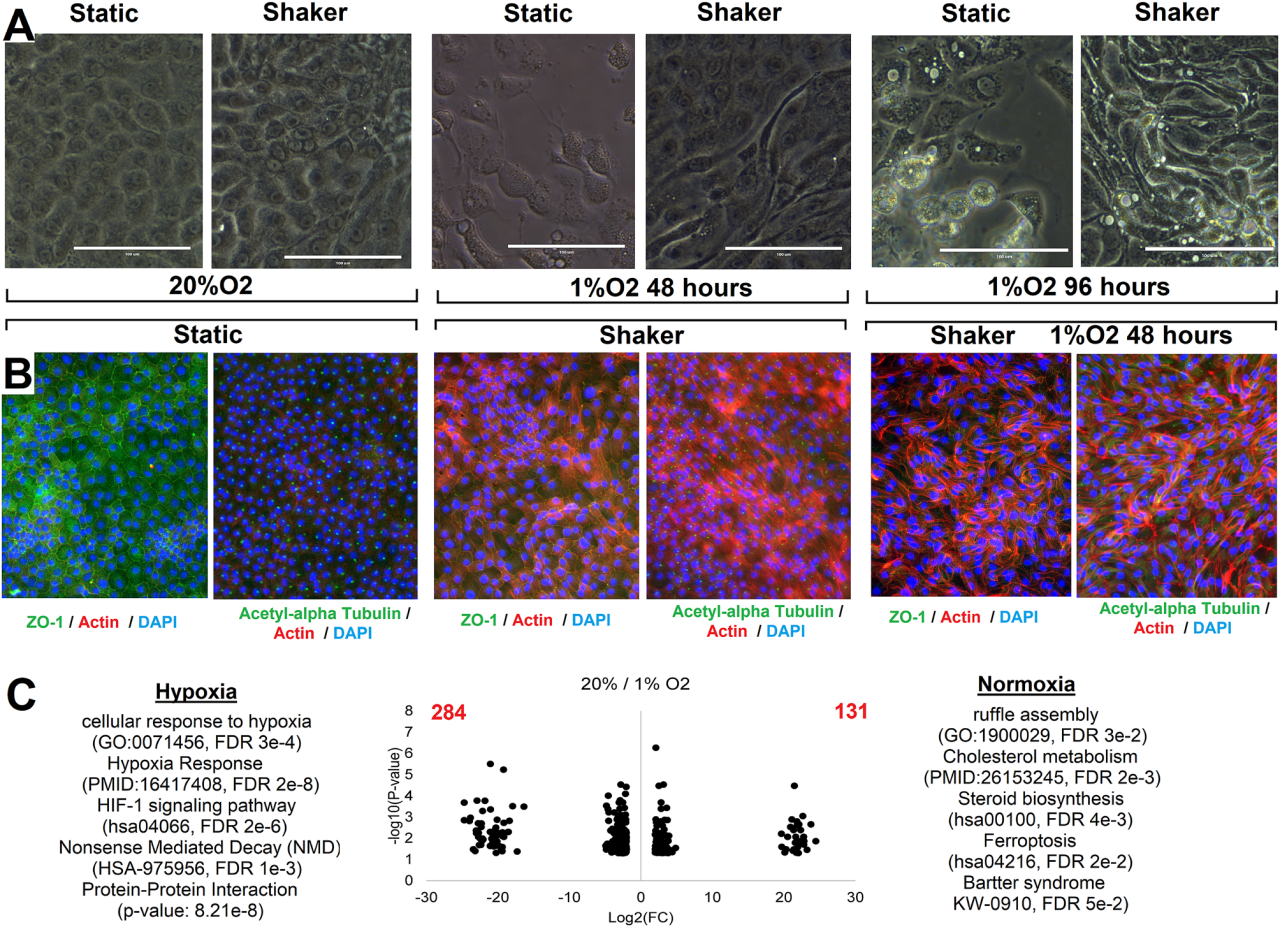
Development of a renal proximal tubule cell culture system using RPTEC-TERT1 cells. (**A**) Light microscope images of RPTEC TERT1 cells under static or shaker conditions in different oxygen concentrations. Note the drawn out structure seen when cells are under hypoxic and shaking conditions. (**B**) Immunofluorescence of different markers in different conditions (ZO1 or aetyl-alpha tubulin in green, Actin in red, nuclei in blue). Actin strands increase and become oriented perpendicular to the fluid flow when under normoxia and constant shaking. Shaking and hypoxia, however, disrupts the cilia and actin strands begin to surround the cells. (**C**) Volcano plot of RNAseq for the log2 of normoxic vs hypoxic conditions, with significant pathways shown on the right and left of the chart.

To build a model of kidney disease in which to study the role of genes involved in kidney injury, specifically *Sept8*, we developed a human renal proximal tubule epithelial cell (RPTEC-TERT1) culture system (Fig. 4). Under normal conditions, RPTEC-TERT1 cells reached confluency and halted proliferation. When the confluent cultures were exposed to hypoxia, however, the cells detached from the surface of the plastic (Fig. 4A), posing a challenge for further study. With continual shaking of the media over the surface of the RPTEC-TERT1 cells for extended time (4 weeks), the cells remained attached to the surface of the plates. When exposed to 1% O_2_ for up to 4 days (96 h), a drawn-out structure typical of cell damage response was seen (Fig. 4A). Morphometric analysis of cultures exposed to hypoxia and shaking reveals a change in cell area, perimeter, circularity, and ellipse radius relative to cells under normal conditions (Figure S2). Using immunofluorescence (IF), cells demonstrated tight junctions (ZO-1) and organized acetyl-alpha tubulin staining common of cilia. When exposed to constant shaking under normoxia there was an increase in actin strands and these became oriented perpendicular to the fluid flow (Fig. 4B). Under shaking and hypoxia, however, acetyl-alpha tubulin became disrupted and the actin strands began to surround the cells, representing altered cellular structure.

RNAseq of the cells under constant shaking vs static conditions revealed minor changes, with an elevation of lysosomal biogenesis transcripts (Figure S3). RNAseq of normoxic vs hypoxic conditions of the cells under constant shaking revealed a higher number of altered transcripts: 284 higher in hypoxia and 131 higher in normoxia (Fig. 4C). We note disruption of multiple pathways expected to be altered in fibrosis and hypoxia including genes associated with HIF-1 signaling and nonsense mediated decay, as well as genes linked to metabolism and biosynthesis, ruffle assemblies, and renal Bartter syndrome that are higher in normoxic conditions. These data support hypoxia, combined with shaking, as a model of RPTEC-TERT1 cell damage.

### Sept8/SEPTIN8 plays a role in kidney damage response

**Figure 5:**
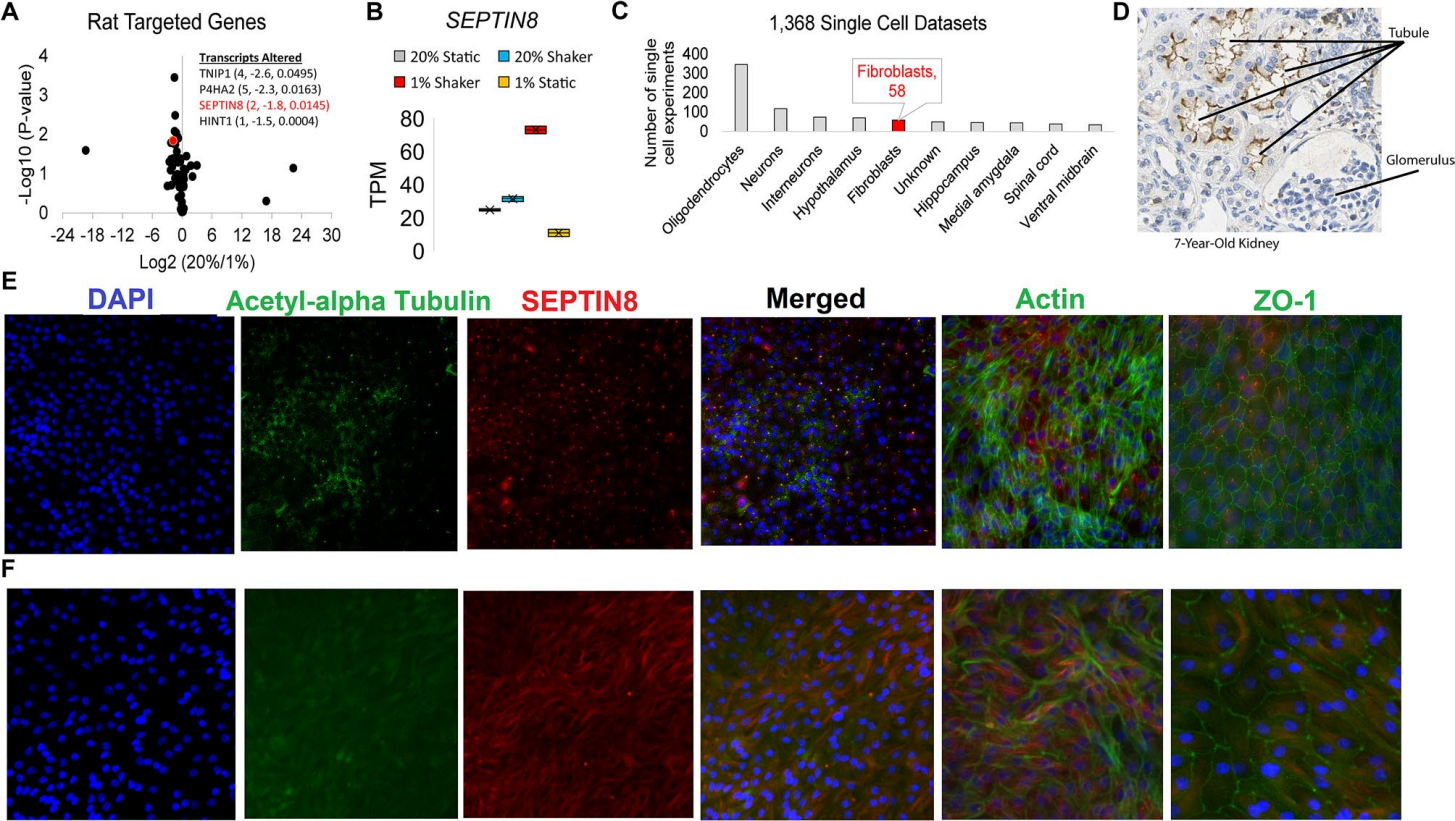
Data implicating *Sept8/*SEPTIN8 in the human tubule hypoxia cell culture model. (**A**) RNAseq values of genes within rat LD block within the RPTEC TERT1 normoxic vs hypoxic conditions. Genes on the right are significant genes. (**B**) TPM values in each of the RNAseq groups for *SEPTIN8* in the RPTEC TERT1 cells. (**C**) Expression of *SEPTIN8* in single cell datasets (human and mouse). (**D**) Immunohistochemistry from the Human Protein Altas (modified from www.proteinatlas.org/ENSG00000164402-SEPT8/tissue/kidney^42^) for SEPTIN8 using HPA005665 antibody. Labels are added for tubules and glomerulus. (**E**) Immunofluorescence of SEPTIN8 (red) with acetyl-alpha tubulin, actin, or ZO-1 (green) as labeled. SEPTIN8 localizes with acetyl-alpha tubulin under shear stress with normoxic conditions. (**F**) Under hypoxic conditions, however, SEPTIN8 re-localizes near the actin filaments.

We queried genes within the rat chromosome 10 UPE locus to identify those that were differentially expressed in the hypoxia model above. We identified four significantly differentially expressed transcripts including *P4HA2* (Fig. 5A) and *SEPTIN8* (Fig. 5A,B), with significantly increased levels of SEPTIN8 under shaking/hypoxia conditions. Using multiple single cell datasets, we found that *SEPTIN8/Sept8* (human and mouse) is frequently expressed in oligodendrocytes, but also shows high expression in fibroblasts (Fig. 5C). We also obtained immunohistology results of a 7-year-old healthy kidney from the human protein atlas (www.proteinatlas.org)^42^. In this healthy kidney, SEPTIN8 is found on the apical surface of proximal tubules, where cilia would protrude, with low concentrations within all other kidney regions (Fig. 5D). Within our tubule model under shear stress, the SEPTIN8 protein localized with acetyl-alpha tubulin (Fig. 5E), suggesting cilia localization and supporting the human immunohistology. Additional staining of SEPTIN8 relative to actin or tight junctions (ZO-1) confirms specificity with acetyl-alpha tubulin colocalization. Upon hypoxic conditions, however, SEPTIN8 becomes more diffuse and localizes to the edges of the cell next to actin filaments (Fig. 5F). We note that there are still some areas where acetyl-alpha-tubulin is speckled in both Figs. 4 and 5, although both figures clearly show more diffuse staining of both acetyl-alpha tubulin and SEPTIN8 under hypoxic conditions. These data suggest a role of SEPTIN8 in structural integrity and cellular response to environmental stress.

**Figure 6:**
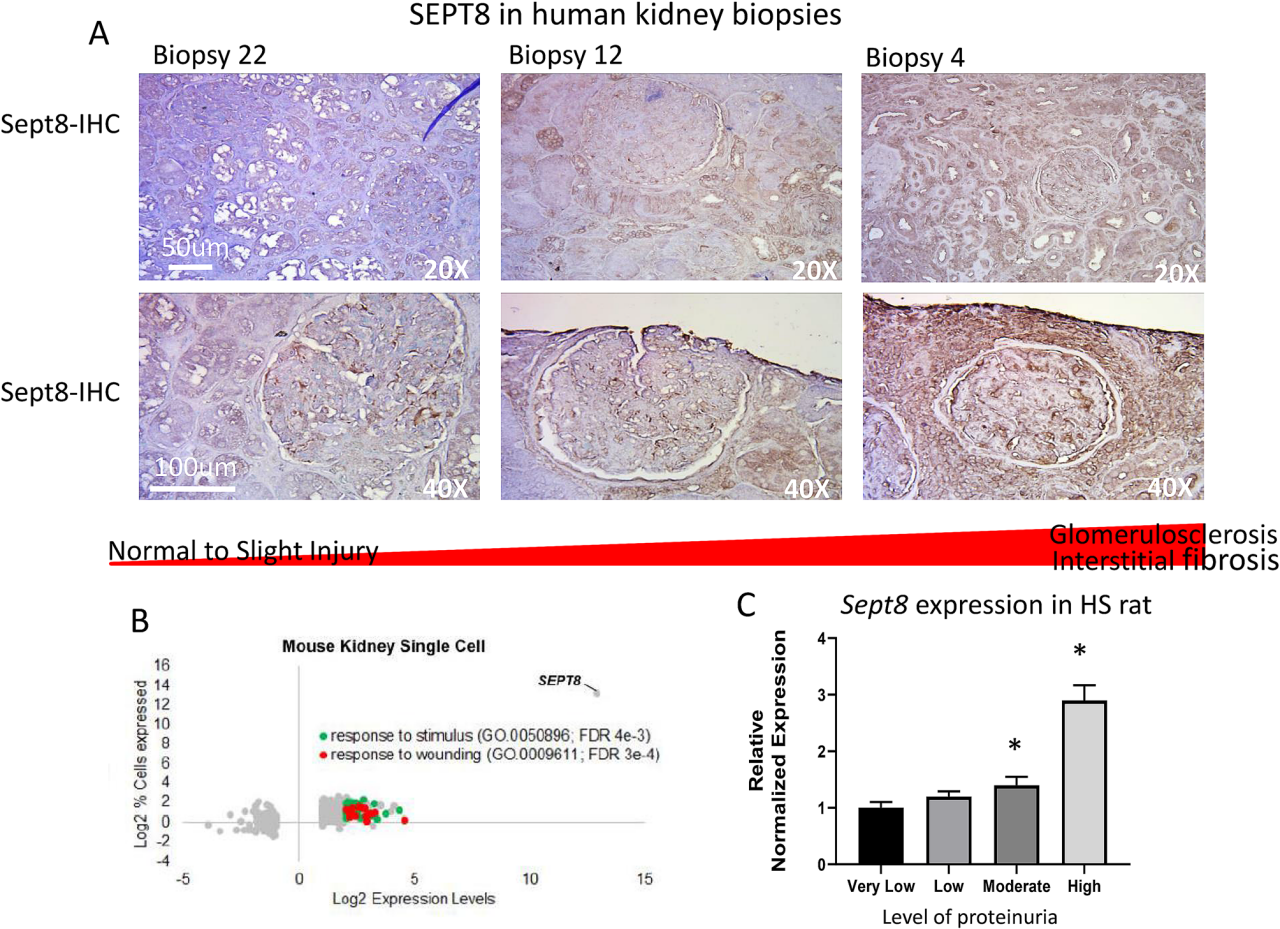
SEPTIN8/*Sept8* expression increases under conditions of fibrosis or wounding. (**A**) Immunohistochemistry of SEPTIN8 in human biopsies. Results are shown from three separate biopsies with varying levels of tubulointerstitial injury ranging from none (biopsy 22) to moderate (biopsy 12) to severe (biopsy 4). The top panel provides representative low resolution (20X) image of the tubule/interstitial region. The bottom panel provides an independent representative high resolution image (40X) of a single glomerulus. (**B**) Interrogating mouse single-cell databases demonstrates very high levels of *Sept8* in response to stimulus or wounding and (**C**) HS rats with high levels of UPE exhibit significantly increased levels of *Sept8* relative to HS rats with low to moderate urinary protein levels. rt-qPCR was run in all HS rats that were selected for histological analysis as described in Fig. 1. *p < 0.05 vs very low, ^†^*p* < 0.0001 vs all groups.

In adult human kidney biopsies, SEPTIN8 expression was observed in multiple areas of the kidney (tubules, glomerulus, small renal vessels) (Fig. 6A). Staining appears to increase with increasing levels of fibrosis, although IHC should not be viewed as quantitative, and thus this should be interpreted with caution^43^. In addition, segregation of genes within mouse single cell datasets^44^ for cells expressing SEPTIN8 elucidated a gene enrichment for stimulus and wounding within the cells (Fig. 6B), further implicating a role of this gene in cellular response to injury and establishing a link between the rat genetic mapping and human model data with mouse in vivo responses. Although *Sept8* did not map as a cis-eQTL to the chromosome 10 locus (likely because relatively few rats that were genotyped had very high UPE levels), we ran rt-qPCR in HS rats selected for histological analysis and found that *Sept8* expression levels increased significantly (one-way ANOVA, *p* < 0.0001) in animals with moderate to high levels of UPE (Fig. 6C). Together, these data strongly support a role of *Sept8* in kidney injury and cellular response to stress.

### HS serum biochemistry genetic mapping

**Figure 7:**
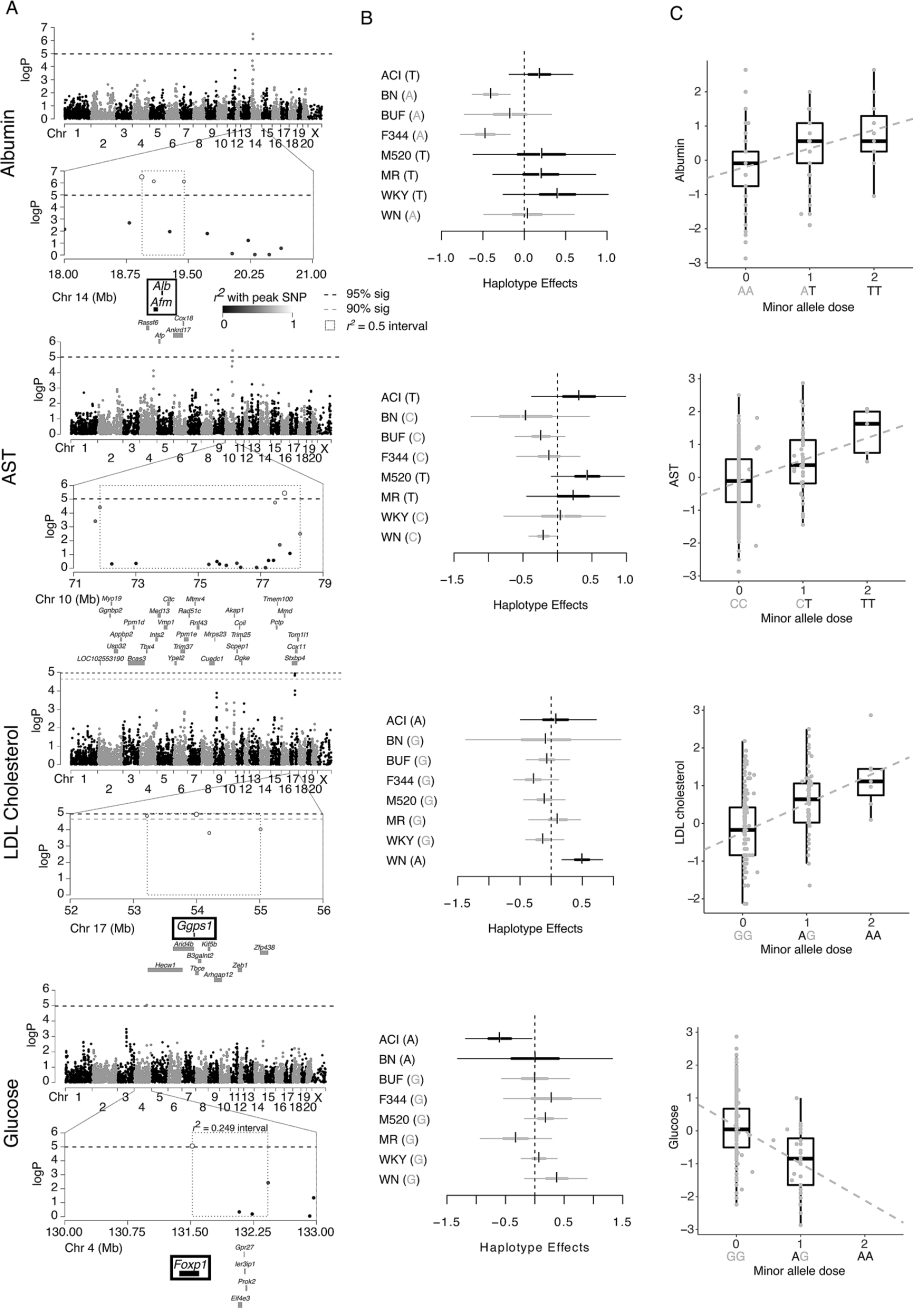
Genome scans for select intermediary serum biochemistry measures. (**A**) Genome scan for select serum biochemistry measures including albumin, AST, LDL cholesterol, and glucose. The x-axis is the position on the chromosome, and the y-axis is the − log_10_ P level of association. Genome-wide significance thresholds were calculated using parametric bootstraps from the null model. The linkage disequilibrium support interval for each QTL is highlighted, showing neighboring markers that were correlated with the peak marker. Annotation of genes that fall within the r^2^ support interval is found underneath the plot. Both *Abm* and *Afm* fall within the albumin QTL and are likely candidate genes. *Foxp1* is directly below the peak association with no other markers in high LD (r^2^ = 0.5) and is the most likely candidate for the glucose QTL. QTL effects as (**B**) additive haplotype effects estimated with the Diploffect model, which takes into account uncertainty in haplotype state, and (**C**) in the actual data, plotted as phenotype vs minor allele dosage of the peak marker. Boxplots represent most likely genotypes.

HS rats exhibited strong variation for multiple serum biochemistry measures that may serve as intermediary phenotypes for CKD (Figure S4). These traits were also strongly heritable (Table 1). We identified significant QTL for several biochemistry measures, including: albumin on rat chromosome 14 (18.94–19.45 Mb, − logP = 6.5, effect size 32%), AST on rat chromosome 10 (71.85–78.27 Mb, − logP = 5.4, effect size 22%), LDL cholesterol on rat chromosome 17 (53.22–55.01 Mb, − logp = 4.9, effect size 20%), and glucose on rat chromosome 4 (131.52–132.42 [r^2^ = 0.249; highest LD with peak marker], − logP = 5.0, effect size 21%) (Fig. 7A). However, no significant associations were observed for serum creatinine or BUN, typical measures of renal function. Genome sequence data from the HS founders, haplotype effects and literature searches were used to identify plausible candidate genes. Founder haplotype effects at the peak marker are shown in Fig. 7B with allele effects shown in Fig. 7C. There were 10 genes within the albumin locus, including albumin (*Abm*) and alpha-albumin (*Afm*). The A allele from BN, BUF, F344, and WN led to decreased albumin levels at this locus. There was a non-synonymous variant that is predicted to be possibly damaging within *Afm* (position 19,082,289 bp) in the founder strains that exhibit the allele effect. The I to M change is likely to alter hydrophobic collapse and is found conserved as a V, I, or L in all vertebrate species, never an M, further implicating *Afm* as the causal gene (Figure S4). There were 23 genes within the locus for LDL cholesterol, only 9 of which are known. Although there was a non-synonymous variant from the WN founder (the founder haplotype that leads to increased LDL cholesterol at this locus) in *Arhgap12*, the variant is neither highly conserved or likely to be damaging. *Ggps1* is another candidate in this region, as it plays a role in the cholesterol biosynthesis pathway (www.rgd.mcw.edu) (Fig. 7A). Within the glucose locus, a single gene, *Foxp1,* falls directly under the peak marker, with four additional genes falling within a more conservative LD interval (r^2^ = 0.249; *Gpr27, Ier3ip1, Prok2, Eif4e3*) (Fig. 7A). None of these genes had a highly conserved non-synonymous variant that is expected to be damaging. *Foxp1* has previously been shown to regulate hepatic glucose homeostasis^45^, and insulin stimulated glucose uptake^46^, making it a strong candidate, although both *Gpr27*^47,48^ and *Prok2*^49^ also play a role in glucose related phenotypes.

## Discussion

The HS rat model provides a unique, highly-recombinant, genetic structure for fine-mapping genetic loci involved in complex traits. This study is the first to utilize the HS model, in conjunction with a novel in vitro model of human tubule damage response, to identify genetic factors associated with kidney injury. Specifically, we identify *Sept8/SEPTIN8* as a strong candidate for kidney injury, demonstrating its role in structural integrity of the cell and response to stress. We also identify additional candidate genes for intermediary serum biochemistry measures linked to CKD.

Using genetic analysis in HS rats, we identify two loci for UPE on chromosomes 2 and 10 as well as loci for several serum biochemistries. To identify candidate genes that may underlie these traits, a number of genomic and bioinformatics tools were utilized. Using these tools, we identify a highly conserved variant predicted to damage protein function within *Sept8,* a gene within the chromosome 10 UPE locus.

To validate the role of *Sept8* in the development of renal injury, we developed a novel human tubule hypoxia-induced damage model using RPTEC-TERT1 cells. Although RPTEC-TERT1 cells have been used to study transport functions, including glucose, ion, or drug transport and to test cytotoxicity of new drugs^50^, their use for studying tubule damage has been challenging because the cells lift off the plate in response to hypoxia. We have found that once exposed to shaking conditions for an extended period of time, however, the cells continue to adhere to the plate even under hypoxic conditions. We show here that RPTEC-TERT1 cells exposed to shaking and hypoxia exhibit both morphological and gene expression changes frequently associated with damage response, thereby validating the model.

Using this in vitro model, we demonstrate two main changes in *SEPTIN8/*SEPTIN8 (mRNA and protein) in response to hypoxia-induced damage: (1) a significant increase in *SEPTIN8* expression levels and (2) a re-localization of SEPTIN8 toward the edge of the cell near actin filaments. As detailed below, these findings are supported by our findings in both the rat model, mouse data-sets, and human kidney biopsies, as well as previous work by others, thereby strongly supporting *Sept8/SEPTIN8* as a renal injury candidate gene involved in kidney damage.

In the current work, *SEPTIN8/Sept8* expression increases in the in vitro model, in mouse single-cell data-sets responding to stimulus or wound, and in HS rats with high proteinuria and kidney fibrosis. These findings support previous work demonstrating increased Septins, including *Sept8/SEPTIN8,* in fibrotic mouse and human kidneys^43^. Although previous work using an experimental unilateral ureter obstruction model found similar renal fibrosis in *Sept8* knock-out mice relative to wild-type mice, a significant upregulation of various other septins were observed in fibrotic kidneys of the *Sept8* knock-out mice compared to contralateral kidneys, indicating likely compensatory mechanisms^43^. Knock-down of *SEPTIN6* (which is similar in sequence homology to *SEPTIN8*) results in decreased fibrosis in hepatic stellate cells^51^, again supporting this possibility. Taken together, results in various species and models, including the current work, demonstrate upregulation of *Setp8/SEPTIN8* in response to cellular damage or fibrosis.

Our data demonstrate that SEPTIN8 is found within tubule cells of the kidney. In RPTEC-TERT1 cells, we show that under normal conditions, SEPTIN8 co-localizes with acetyl-alpha tubulin, indicative of cilia localization. This is supported by immunohistology of a young, healthy human kidney, with SEPTIN8 localizing to the apical surface of proximal tubules where cilia protrude. IHC of adult kidneys show more generalized staining of SEPTIN8 within the kidney, indicating more work is needed to determine exact location of this protein within the kidney. Previous work, however, has shown that SEPTIN8 localizes to ciliary sub-compartments within lung epithelium^52^, further supporting potential ciliary localization of this gene. In the current work, we demonstrate that when the tubule culture system is under hypoxia, SEPTIN8 staining becomes more diffuse and localizes to the edges of the cell next to actin filaments. It should be noted that some speckling staining of SEPTIN8 is still found in hypoxia cultures, but this is minor relative to staining seen at the edge of the cell. These data suggest an important role of SEPTIN8 in structural integrity of the cell.

The current work does not lend itself to a full understanding of how *Sept8*/SEPTIN8 leads to cytoskeletal changes and eventual increases in UPE. One specific limitation of the current work is that we were unable to successfully knock-down SEPTIN8 in the cell culture system; studies that would prove invaluable in further supporting a role of this gene and in understanding its function in kidney damage. That said, the current work, combined with what is known in the literature, can provide some clues. Septins are GTPase proteins that are co-expressed with extracellular matrix components and crucial for cytoskeleton organization. Septins have been shown to play a role in multiple functions including membrane remodeling and compartmentalization, cytoskeleton rearrangement, vesicle trafficking, and apoptosis (see^43,53^). Fibrosis is marked by an excess of extracellular matrix protein in tubuloinsterstitial compartments, myofibroblast transformation, and cytoskeletal rearrangement^54^. Several ciliopathies, including polycystic kidney disease, exhibit fibrosis^55^. In addition, primary cilia are involved in myofibroblast transition^56^, a key process in fibrosis. Potential ciliary localization of SEPT8 under normal conditions with re-localization toward actin filaments during cellular damage suggests *Sept8/SEPTIN8* may play a role in cell damage through myofibroblast transition and/or cytoskeleton reorganization. In addition, previous work has found that SEPTIN8 partners with mitogen-activated protein kinase 5 (MK5)^53^, pointing to its role in vesicle trafficking. In neuroblastoma cells, M5K phosphorylates SEPTIN8 and this could account for the rapid re-localization of SEPTIN8 under hypoxic conditions in the current study. It should also be noted that MK5 has been connected to cellular stress, hypoxia-induced cell migration, and fibrosis pathways^57^. Given the current data, we thus propose that under normal conditions, *Sept8/SEPTIN8* functions at the apical surface and is likely involved in endocytic vesicle trafficking. Upon cellular stress, however, SEPTIN8 relocalizes near actin filaments and may become part of the stress fibers of hypoxia induced migration and/or myofibroblast transition, eventually leading to fibrosis. Importantly, the current studies are only the beginning of trying to understand function of SEPTIN8 and much more work is needed to test these hypotheses.

Another limitation of this work is that the current studies are unable to unravel the relative contributions of the V102M coding variant (which is protective against proteinuria) and unidentified regulatory variants altering expression levels (which increase proteinuria susceptibility) within *Sept8* on kidney fibrosis and proteinuria. The current work in the HS rat, combined with functional analysis using the in vitro hypoxia model, however, suggest that subtle changes in genetic variation within *Sept8/SEPTIN8* may interact with other disease factors (eg, age, hypertension, stress, and glomerular damage) to alter susceptibility to kidney fibrosis. Based on the above, we postulate that increased *Sept8* expression in the kidney leads to increased cell migration and eventual fibrosis, although it is important to note that the current studies are unable to determine if increased *Sept8* expression is a cause or consequence of tubule damage. In contrast, we postulate that subtle change in *SEPT8* function via the V102M variant prohibit *Sept8* re-localization and thus protect against fibrosis and increased UPE. These interactions will be explored in future studies.

While most groups have studied genetics of the glomerulus through either podocyte or endothelial dysfunction, several mapping studies in the rat suggest tubule genetics also contribute to CKD, with the work here further supporting these contributions. This mechanism is likely through initial glomerular damage that propagates into altering the tubule and interstitial space, with genetics elevating the damage response, fibrosis, and progression into CKD^13,14^. Our new tubule model system is a potential powerful model to test the genetics that contribute to this mechanism. Future work in HS rats with the V102M mutation will also enable us to test this hypothesis.

In contrast to the chromosome 10 region, we are unable to find a plausible candidate gene within the chromosome 2 UPE locus despite the fact that it is only 0.2 Mb and contains a single gene, *Pyruvate dehydrogenase E1 Alpha 2 subunit (Pdha2).* This gene is involved in pyruvate metabolism and the Hif-1 signaling pathway (www.genecards.com), although no work has previously linked it to kidney disease. Although *Pdha2* possesses a cis-eQTL in the chromosome 2 region, it is expressed at very low levels in the kidney. Furthermore, there is no correlation between *Pdha2* expression levels and urinary protein, and the gene is not supported using mediation analysis. None of the genes in the surrounding region (*Bmpr1b, Pdlim5, Stpg2*, and *Unc5c*) possess cis-eQTLs, are correlated with urinary protein levels, or have highly conserved potentially damaging variants within the WKY founder strain. Together, these data indicate that a variant within this region may be driving a gene or genes that fall outside of the chromosome 2 locus or falls within an unknown genetic element (eg, miRNA). Further analysis, such as global gene expression to identify trans-eQTLs and/or chromosome confirmation capture^58^, will be needed to determine genes driving the phenotype at this locus.

In addition to identification of *Sept8* as the likely causal gene within the chromosome 10 UPE locus, *Foxp1* (*Forkhead Box P1*) was identified as a candidate within the glucose locus on rat chromosome 4 and *Afm* (*Afamin* of *Alpha-albumin*) as a candidate within the albumin locus on rat chromosome 14. Both glucose and albumin may serve as important intermediate traits for CKD. Diabetes is the leading cause of CKD^59^ and serum albumin is associated with higher risk of incident end stage renal disease (ESRD) independent of baseline urine albumin to creatinine ratio and other ESRD risk factors^60^. We found that *Afm* contains a highly conserved variant that is predicted to be damaging in the founder strains with the allelic effect on the phenotype. Although no variants were found in *Foxp1*, this gene falls directly under the peak marker and *Foxp1* has been shown to regulate hepatic glucose homeostasis^45^ and insulin stimulated glucose uptake^46^. Both *Gpr27*^47,48^ and *Prok2*^49^, which fall within the wider confidence interval are also potential candidates within this region. No significant associations were observed for serum creatinine and BUN. This is expected given the relatively modest level of renal injury (eg, few rats with pathological levels of proteinuria) which is likely not sufficient to reflect significant changes in renal function.

Because the current GWAS was conducted using only 245 HS male rats, we were surprised at the high level of success in identifying genetic loci. This success is likely attributed to the fact that the QTL identified explain a large percentage of the variance (~ 20%) of each trait. Previous work in our laboratory has demonstrated that a sample size of 700 is sufficient to identify QTL that explain ~ 10% of the variance^21^. Other studies, however, have used over 1000 HS animals, and this tends to be sufficient to identify QTL that explain less than 10% of the variance^61,62^. Simulations by our group have demonstrated that increased sample size leads to an exponential increase in the number of QTL identified (unpublished), indicating that increasing sample size will likely lead to additional loci for urinary protein and serum biochemistries in the HS rat.

The current work has used HS rats and a novel human tubule hypoxia induced damage cell culture model to identify *Sept8* as the likely causal gene underlying a locus for UPE. We demonstrate a role of *Sept8/SEPTIN8* in cell structure and integrity and demonstrate changes in the gene in response to environmental perturbation. This work validates, for the first time, an in vitro model of kidney tubule damage and highlights the utility of this, in combination with genetic mapping in HS rats, for identifying novel gene regulators that drive kidney disease.

## Supplementary Information

Supplementary Information.
