# Supplementary material for: Sept8/SEPTIN8 involvement in cellular structure and kidney damage is identified by genetic mapping and a novel human tubule hypoxic model
Source: Sci Rep. 2021 Jan 22;11:2071. doi: 10.1038/s41598-021-81550-8 (PMC7822875; doi:10.1038/s41598-021-81550-8)
Supplement: Supplementary file 1 — Supplementary Information. [file 41598_2021_81550_MOESM1_ESM.pdf]

Supplementary Information

Sept8/SEPTIN8 involvement in cellular structure and kidney damage is identified by genetic mapping and a novel human tubule hypoxic model

Gregory R. Keele[1+], Jeremy W. Prokop[2,3+], Hong He[4], Katie Holl[4], John Littrell[4], Aaron W. Deal[5], Yunjung Kim[6], Patrick B. Kyle[8], Esinam Attipoe[8], Ashley C. Johnson[8], Katie L. Uhl[3], Olivia L. Sirpilla[3], Seyedehameneh Jahanbakhsh[3], Melanie Robinson[2], Shawn Levy[2], William Valdar[6,7], Michael R. Garrett[8*], Leah C. Solberg Woods[5*]

[1]The Jackson Laboratory, Bar Harbor, ME, [2]HudsonAlpha Institute, Huntsville, AL, [3]Department of Pediatrics and Human Development, Department of Pharmacology, Michigan State University, Grand Rapids, MI, [4]Medical College of Wisconsin, Departments of Pediatrics and Physiology, Milwaukee, WI, [5]Wake Forest School of Medicine, Department of Internal Medicine, Winston Salem, NC, [6]Department of Genetics and [7]Lineberger Comprehensive Cancer Center, University of North Carolina at Chapel Hill, Chapel Hill, NC, [8]Department of Pharmacology, Medicine (Nephrology), Pediatrics (Genetics), University of Mississippi Medical Center [+]These authors contributed equally as first authors *These authors contributed equally to this study as senior authors.

Supplemental Figure Legends

Figure S1. Representative histological injury in the HS kidney for low and high UPE.

Figure S2. Morphology analysis of RPTEC-TERT1 cells under shaking conditions with or without hypoxia. A) Light images of 20% O2 normoxic RPTEC-TERT1 cells, with 1% O2 hypoxic for 48 hours, or 1% O2 for 96 hours. B) Colored segmentation map from MorphoLibJ. C) Cell trace from MorphoLibJ. D) Quantitative analysis of segmentation performed in MorphoLibJ.

Figure S3. RNAseq of cells under constant shaking vs a static condition.

Figure S4. HS rats exhibit large variation for select biochemistry measures including A) albumin (mg/dl), B) AST (mg/dl), C) glucose (mg/dl) and D) LDL cholesterol (mg/dl). The thick black horizontal line indicates the median with the blue lines denotes 25% and 75% quartiles, respectively**.**

Figure S5. Protein model of the I485M amino acid change within *Afm.*

Supplemental Table S1: Genes and target probes used for targeted RNAseq within Chrs. 2 and 10.

Figure S1. Representative Histological Injury in the HS Kidney for Low and High UPE

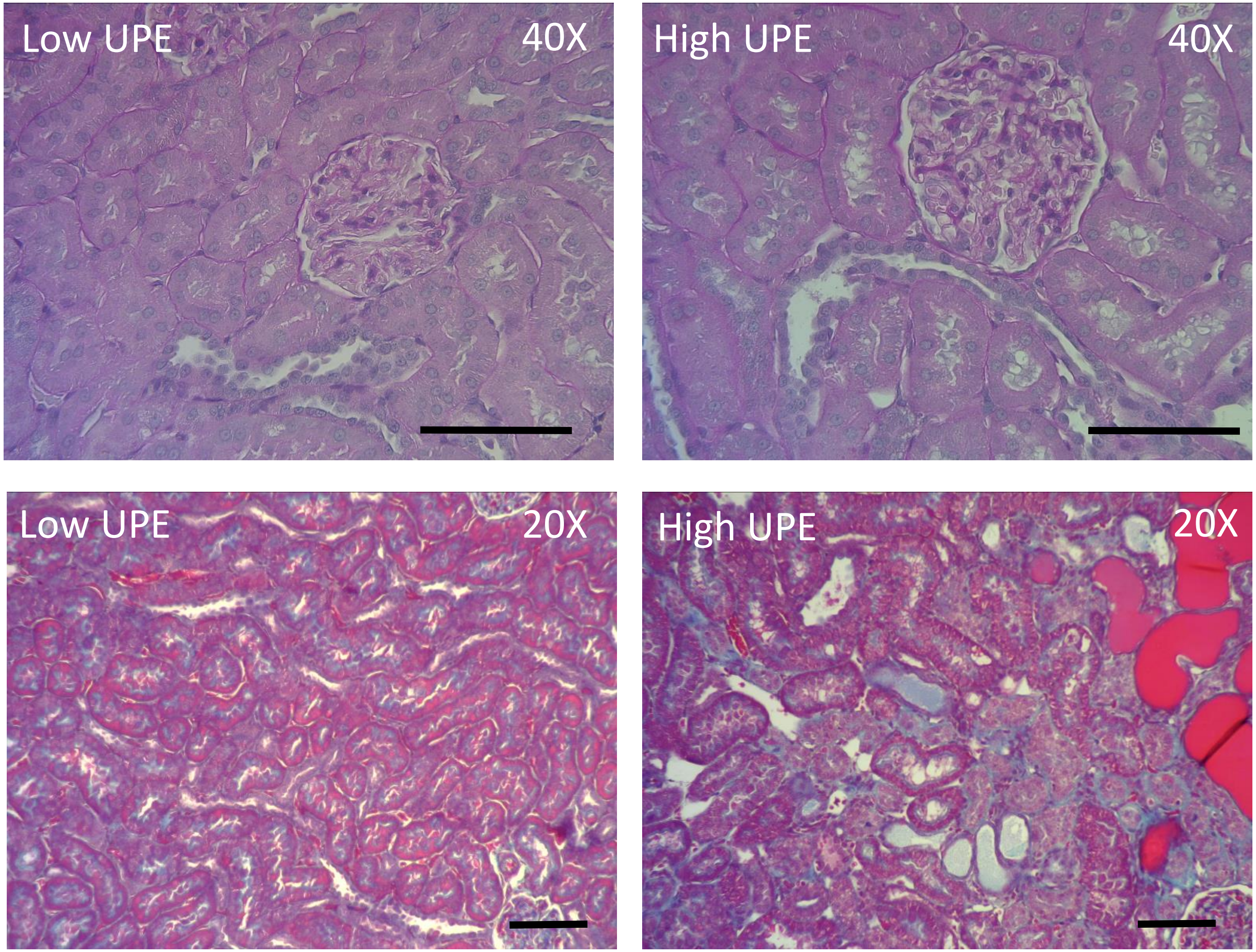

Figure S2. Morphology analysis of RPTEC-TERT1 cells under shaking conditions, with and without hypoxia

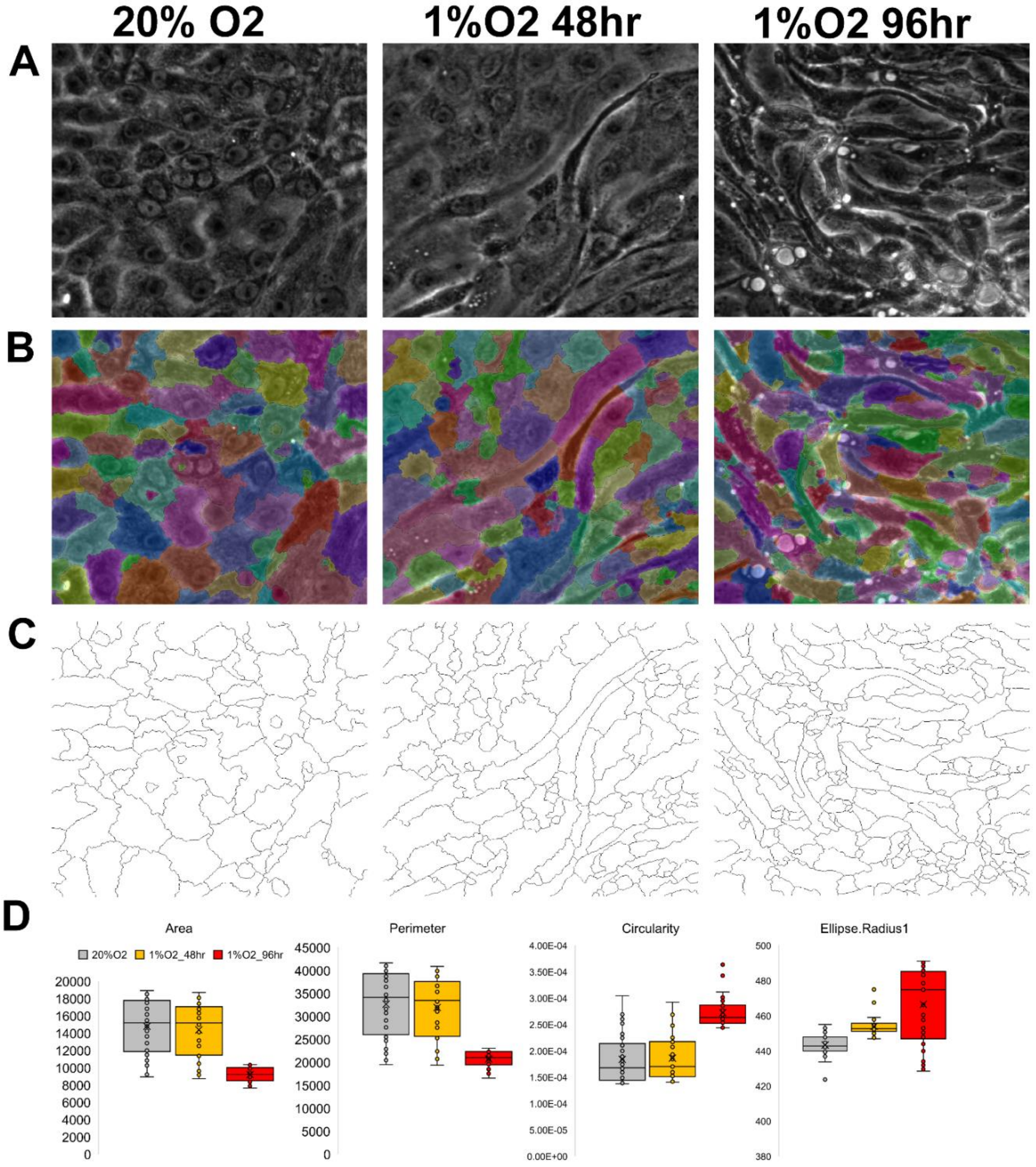

Figure S3. RNAseq of cells under constant shaking vs a static condition

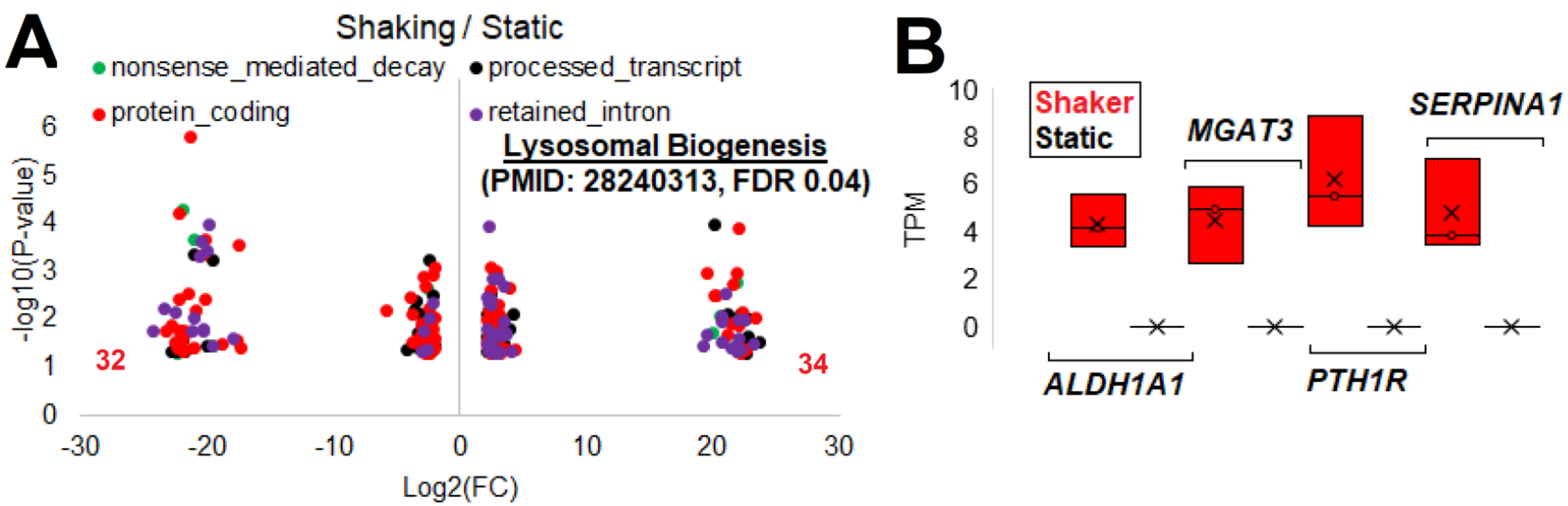

Figure S4. Distribution of select biochemistry measures in male HS rats

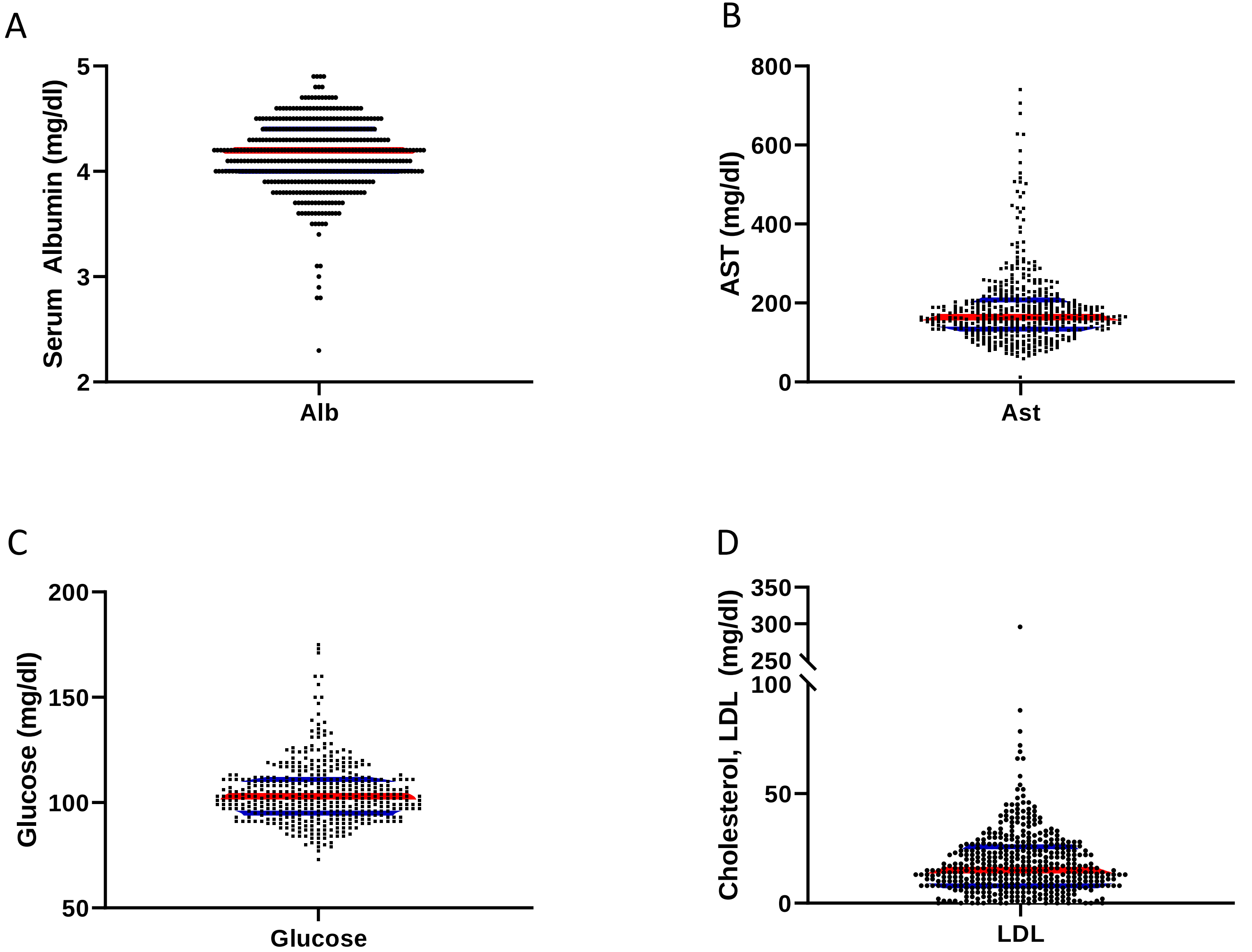

Figure S5: Protein model of the I485M amino acid change within *Afm.*

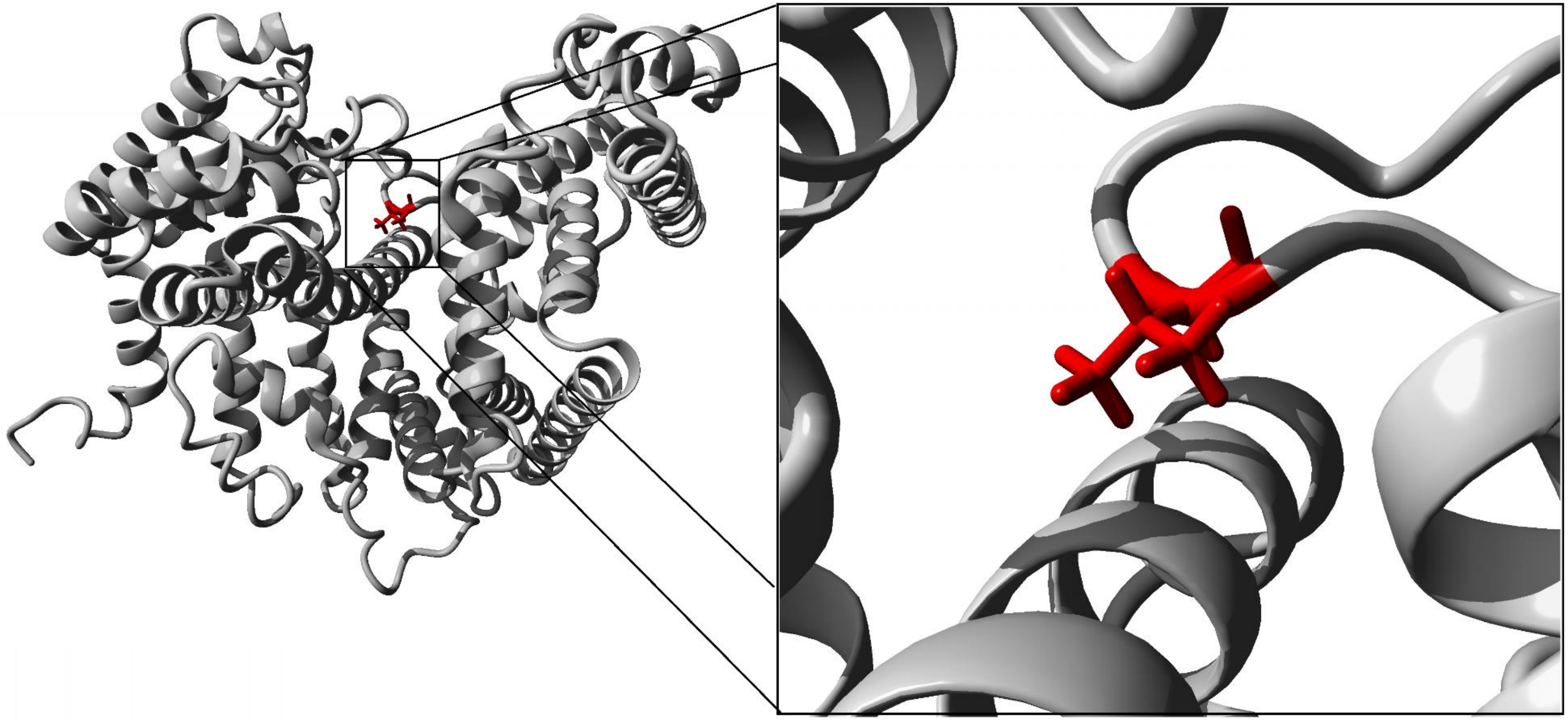

Supplemental Table S1

Chromosome	Gene Name	Transcript ID	Assay ID	Left Exon	Right Exon	Build ID	Start Position
chr2	Bmpr1b	ENSRNOT00000021963	12006208	6	7	rn6	247423412
chr2	Bmpr1b	ENSRNOT00000021963	12006209	8	9	rn6	247406185
chr2	Pdha2	ENSRNOT00000021719	12006242	0	0	rn6	246736784
chr2	Pdha2	ENSRNOT00000021719	12006243	0	0	rn6	246737776
chr2	Pdlim5	ENSRNOT00000022387	12006244	1	2	rn6	247985435
chr2	Pdlim5	ENSRNOT00000022387	12006245	5	6	rn6	247883558
chr2	Stpg2	ENSRNOT00000029382	12006260	10	11	rn6	244752128
chr2	Stpg2	ENSRNOT00000029382	12006261	12	13	rn6	245040155
chr2	Unc5c	ENSRNOT00000082287	12006264	6	7	rn6	247351664
chr2	Unc5c	ENSRNOT00000082287	12006265	12	13	rn6	247381012
chr10	Acsl6	ENSRNOT00000058817	12006204	13	14	rn6	39694599
chr10	Acsl6	ENSRNOT00000058817	12006205	19	20	rn6	39707980
chr10	Aff4	ENSRNOT00000009440	12006206	5	6	rn6	38740178
chr10	Aff4	ENSRNOT00000009440	12006207	19	20	rn6	38763006
chr10	Cdkl3	ENSRNOT00000079411	12006210	2	3	rn6	37466206
chr10	Cdkl3	ENSRNOT00000079411	12006211	6	7	rn6	37481192
chr10	Cdkn2aipnl	ENSRNOT00000006708	12006212	0	1	rn6	37422864
chr10	Cdkn2aipnl	ENSRNOT00000006708	12006213	1	2	rn6	37425006
chr10	Fnip1	ENSRNOT00000012671	12006214	8	9	rn6	39855961
chr10	Fnip1	ENSRNOT00000012671	12006215	10	11	rn6	39865205
chr10	Fstl4	ENSRNOT00000067809	12006216	5	6	rn6	38419494
chr10	Fstl4	ENSRNOT00000067809	12006217	12	13	rn6	38521239
chr10	Gdf9	ENSRNOT00000009727	12006218	0	1	rn6	38789361
chr10	Gpx3	ENSRNOT00000079830	12006219	2	3	rn6	40252142
chr10	Gpx3	ENSRNOT00000079830	12006220	3	4	rn6	40253670
chr10	Hint1	ENSRNOT00000000772	12006221	0	1	rn6	40208379
chr10	Hint1	ENSRNOT00000000772	12006222	1	2	rn6	40211397
chr10	Hspa4	ENSRNOT00000023628	12006223	6	7	rn6	38622810
chr10	Hspa4	ENSRNOT00000023628	12006224	17	18	rn6	38603958
chr10	Il13	ENSRNOT00000010121	12006225	1	2	rn6	38984047
chr10	Il13	ENSRNOT00000010121	12006226	2	3	rn6	38983367

chr10	Il3	ENSRNOT00000031762	12006228	3	4	rn6	39620870
chr10	Il4	ENSRNOT00000010029	12006229	1	2	rn6	38969062
chr10	Il4	ENSRNOT00000010029	12006230	2	3	rn6	38965137
chr10	Irf1	ENSRNOT00000010968	12006231	3	4	rn6	39113146
chr10	Irf1	ENSRNOT00000010968	12006232	7	8	rn6	39115256
chr10	Jade2	ENSRNOT00000082585	12006233	2	3	rn6	37290951
chr10	Jade2	ENSRNOT00000082585	12006234	9	10	rn6	37272059
chr10	Kif3a	ENSRNOT00000077569	12006235	7	8	rn6	38936215
chr10	Kif3a	ENSRNOT00000077569	12006236	14	15	rn6	38949346
chr10	Leap2	ENSRNOT00000049820	12006237	0	1	rn6	38774394
chr10	Lyrm7	ENSRNOT00000075311	12006238	2	3	rn6	40195314
chr10	Lyrm7	ENSRNOT00000075311	12006239	3	4	rn6	40193191
chr10	P4ha2	ENSRNOT00000042144	12006240	5	6	rn6	39447952
chr10	P4ha2	ENSRNOT00000042144	12006241	14	15	rn6	39462036
chr10	Ppp2ca	ENSRNOT00000007621	12006246	1	2	rn6	37542390
chr10	Ppp2ca	ENSRNOT00000007621	12006247	5	6	rn6	37537429
chr10	Rad50	ENSRNOT00000063772	12006248	18	19	rn6	39026088
chr10	Rad50	ENSRNOT00000063772	12006249	23	24	rn6	39004098
chr10	Rapgef6	ENSRNOT00000013481	12006250	6	7	rn6	39953097
chr10	Rapgef6	ENSRNOT00000013481	12006251	25	26	rn6	40035286
chr10	Sept8	ENSRNOT00000065229	12006252	3	4	rn6	38891747
chr10	Sept8	ENSRNOT00000065229	12006253	8	9	rn6	38894531
chr10	Shroom1	ENSRNOT00000074769	12006254	3	4	rn6	38822513
chr10	Shroom1	ENSRNOT00000074769	12006255	6	7	rn6	38823846
chr10	Skp1	ENSRNOT00000085463	12006256	1	2	rn6	37599697
chr10	Skp1	ENSRNOT00000085463	12006257	4	5	rn6	37607932
chr10	Slc22a5	ENSRNOT00000081898	12006258	4	5	rn6	39210027
chr10	Slc22a5	ENSRNOT00000081898	12006259	8	9	rn6	39202785
chr10	Tnip1	ENSRNOT00000013864	12006262	6	7	rn6	40270375
chr10	Tnip1	ENSRNOT00000013864	12006263	15	16	rn6	40257624
chr10	Uqcrq	ENSRNOT00000073964	12006266	0	1	rn6	38782153
chr10	Vdac1	ENSRNOT00000008477	12006267	4	5	rn6	37740112
chr10	Vdac1	ENSRNOT00000008477	12006268	7	8	rn6	37750926
chr10	Zcchc10	ENSRNOT00000009098	12006269	3	4	rn6	38668182
